# Effect of electric field on optoelectronic properties of indiene monolayer for photoelectric nanodevices

**DOI:** 10.1038/s41598-019-53631-2

**Published:** 2019-11-21

**Authors:** Deobrat Singh, Sanjeev K. Gupta, Igor Lukačević, Matko Mužević, Yogesh Sonvane, Rajeev Ahuja

**Affiliations:** 1 Condensed Matter Theory Group, Department of Physics and Astronomy, Uppsala University, Box 516, 77120 Uppsala, Sweden; 2 Computational Materials and Nanoscience Group, Department of Physics and Electronics, St. Xavier’s College, Ahmedabad, 380009 India; 3 Department of Physics, Josip Juraj Strossmayer University of Osijek, 31000 Osijek, Croatia; 4 Advanced Material Lab, Department of Applied Physics, S.V. National Institute of Technology, Surat, India; 5 Applied Materials, Department of Materials and Engineering, Royal Institute of Technology (KTH), S-100 44 Stockholm, Sweden

**Keywords:** Electronic structure, Optical materials and structures

## Abstract

In recent years, layered materials display interesting properties and the quest for new sorts of two-dimensional (2D) structures is a significance for future device manufacture. In this paper, we study electronic and optical properties of 2D indiene allotropes with planar and buckled structures. The optical properties calculations are based on density functional theory (DFT) simulations including in-plane and out-of-plane directions of light polarization. We indicate that the optical properties such as complex refractive index, absorption spectrum, electron energy loss function (EELS), reflectivity and optical conductivity spectra are strongly dependent on the direction of light’s polarization. High values and narrow peaks in optical spectra introduce indiene to the field of ultra-thin optical systems. The effect of external static electric field on electronic and optical properties of indiene is also observed and discussed. We show that the band gap in buckled indiene can be effectively changed by applying the external electric field. The discoveries here expand the group of 2D materials beyond graphene and transition metal dichalcogenides (TMDs) and give valuable data for future experimental realization of new mono-elemental materials with conceivable applications in optical devices.

## Introduction

Nowadays, 2D mono-elemental materials beyond graphene^1^ have great interest in the field of nanoelectronics and nano-optics applications. In the recent year, lots of 2D materials such as silicene^2^, antimonene^3,4^, boron-nitride nanosheets^5^, TMDs^6–10^, and black phosphorus^11^ have been theoretically and experimentally synthesized which have extraordinary physical and chemical properties and they are different from their bulk counterparts. Due to the different atomic arrangements of these discovered 2D materials represents metallic, semiconductor and insulator and instead of this some materials have topological insulating properties with very high carrier mobility^12^. Due to the different behaviours of these 2D materials, it have various promising applications such as optoelectronics, photonics, and nanoelectronics, for example, FETs, photovoltaic solar cells applications^6,13–15^.

As functional layers fabricated from thin films and monolayers tend to have distinctively different features from their bulk forms in their electronic band structures, optical properties are expected to be quite different also, and must be investigated on their own. In case of the optical properties, we need to deals with the isolated 2D layer of honeycomb crystal structure and mixture of different materials^16^. For this the thickness, d of layer, must be inspected in the point of confinement as d → 0 which is made by macroscopic estimation procedure, for example, the optical reflection, transmission or ingestion spectroscopy. The macroscopic dielectric function for such type of layered materials can’t characterised from a result point of view. It is previously studied in case of graphene^17^, the finite thickness is possible such as d = 0.3 nm or 0.7 nm. Another experimental issue is the suspicion of an immaterial impression of a one atomic layer thick framework. At that point, an absorbance of such a layer can be characterized, which, in the example of graphene, the 2.3% in the range very near from infrared to visible spectrum as a reference for universal values^18,19^. Here, the optical conductivity of the graphene as a framework was theoretically analysed to maintain the crucial distance for thickness problem^18,20^ after that its spectra characterize by experimentally^18,19^. Thus, the optical conductivity can be utilized to compute the optical properties of such type of 2D layered materials^21^. According to that, we can easily determine the optical conductivity of electrons and holes of Dirac-cone like layered 2D materials such as graphene^19^. Most cases, supercell technique based on first-principles calculations is used to calculates the properties of 2D single layered materials^22^.

In the previous work, the structural and electronic properties of indiene allotropes: planar and buckled geometries of 2D indium^23^ has been studied based on density functional theory (DFT). Also, indium tin oxide (ITO) is the key components of indium and is a heavily doped n-type semiconductor with large band gap around 4 eV^24^ which is widely used in research as well as industry for examples; polymer-based gadgets, thin film photovoltaics, and structural windows etc. Additionally, it was reported that the ITO is also beneficial for some more applications like anti-reflective coatings and transparent electrodes^25^. As previously reported that the ITO is transparent materials in the visible regime, precise (experimental) determination of the absorption is challenging. Therefore, it is important to simulate the optical spectra using *ab initio* theoretical approaches in order to lay the theoretical ground which future experimental data can be built on. Our goal is to give theoretical interpretation of the optical properties of predicted stable indium monolayers. This motivation is driven by two facts: (i) monolayer structures present new pathways in ultra-thin optical devices, and (ii) buckled indiene has shown significant potential with easily tunable band gap under external strain^23^. Furthermore, this study intends to prove that indium allotropes preserve their beneficial optical properties even in atomic thickness limit.

Motivated by above work on the indium-based materials, we computed the structural and electronic properties of planar and buckled indiene. The electronic band structures show that planar indiene has metallic, while buckled indiene has semiconducting nature and it has a band gap of 1.60 eV. Based on these results, we calculate the frequency dependent optical properties and demonstrate anisotropic behaviour. The semiconducting/metallic properties of indiene recommend that this new material may be helpful for flexible electronic, due to ultra-thin structure, and optical applications which may hold the key towards single-layer-material based optics.

## Computational Methods

The electronic structure simulations of indiene monolayer were calculated based on the density functional theory (DFT). Vienna ab-initio simulation package (VASP) code was used for electronic structures calculations, which implements a solution of Kohn-Sham equations in a plane-wave basis set^26^. The projector‐augmented‐wave (PAW) method were used for electron-ion interactions, while the exchange-correlation energy were treated within the generalized gradient approximation (GGA) in the form of Perdew, Burke, and Ernzerhof (PBE) functional^27^. The hybrid functionals based on Heyd‐Scuseria‐Ernzerhof (HSE06) exchange-correlation interactions was adopted to calculate the frequency dependent complex dielectric function and optical properties just as to address the well‐known underestimation of band-gap in PBE estimation^28,29^. The random phase approximation (RPA) was applied^30,31^ during the optical properties. A plane‐wave energy cut-off was set to 400 eV for all the calculations. A vacuum layer with a thickness at least 15 Å was set to prevent physical interactions between the periodic images. A K-points sampling under Monkhorst-Pack meshes of 11x11x1 were used for the first Brillouin zone during the geometry optimizations of monolayers^32^. During the atomic relaxations, all the forces on each atom were smaller than and change of total energies were converged to 0.01 eV/Å and 10^−6^ eV, respectively.

For the frequency dependent optical properties calculations, we taken only interband transitions into account. The frequency dependent complex dielectric function $\varepsilon (\omega )$ were computed in the photon energy range of 0 to 10 eV. Optical properties of any materials are in general computed within the frequency dependent dielectric function: $\varepsilon (\omega )={\varepsilon }_{r}(\omega )+i{\varepsilon }_{i}(\omega )$. The calculations of imaginary part as described earlier using time dependent perturbation theory (TDPT) with dipole approximation. The dielectric matrix has been resolved in the wake of getting the electronic ground state. In the long-wavelength limit (q → 0), the imaginary part of the dielectric function is presented by following relation:

1 $${\varepsilon }_{i{\boldsymbol{,}}{\boldsymbol{\alpha }}{\boldsymbol{\beta }}}({\boldsymbol{\omega }})=\frac{4{{\boldsymbol{\pi }}}^{2}{{\boldsymbol{e}}}^{2}}{\varOmega }\mathop{\mathrm{lim}}\limits_{q\to 0}\frac{1}{{{\boldsymbol{q}}}^{2}}\sum _{{\boldsymbol{c}},{\boldsymbol{v}},{\boldsymbol{k}}}2{{\boldsymbol{w}}}_{{\boldsymbol{k}}}{\boldsymbol{\delta }}({\varepsilon }_{{\boldsymbol{ck}}}-{\varepsilon }_{{\boldsymbol{vk}}}-{\boldsymbol{\omega }})\langle {{\boldsymbol{u}}}_{{\boldsymbol{ck}}+{{\boldsymbol{e}}}_{{\boldsymbol{\alpha }}}{\boldsymbol{q}}}|{{\boldsymbol{u}}}_{{\boldsymbol{vk}}}\rangle {\langle {{\boldsymbol{u}}}_{{\boldsymbol{ck}}+{{\boldsymbol{e}}}_{\beta }{\boldsymbol{q}}}|{{\boldsymbol{u}}}_{{\boldsymbol{vk}}}\rangle }^{\ast },$$

where, ω shows the frequency of the electromagnetic radiation in energy units, Ω is the volume of the supercell, the indices *c* shows conduction band and *v* refer as the valence band, and *u*_*c****k***_ represents the cell periodic part of the orbitals at the reciprocal space point **k**. The real part and the imaginary part of *ε*(*ω*) are connected through the Kramers-Kronig (KK) relation. The real part of the dielectric tensor *ε*_*r*_ is obtained using the KK relation in the form^30,33,34^,

2 $${\varepsilon }_{{\boldsymbol{r}},{\boldsymbol{\alpha }}{\boldsymbol{\beta }}}({\boldsymbol{\omega }})={1}+\frac{{2}}{{\boldsymbol{\pi }}}{\boldsymbol{P}}{\int }_{0}^{\infty }\frac{{\varepsilon }_{{\boldsymbol{\alpha }}{\boldsymbol{\beta }}}^{{2}}({\boldsymbol{\omega }}{\boldsymbol{^{\prime} }}){\boldsymbol{\omega }}{\boldsymbol{^{\prime} }}}{{{\boldsymbol{\omega }}{\boldsymbol{^{\prime} }}}^{{2}}-{{\boldsymbol{\omega }}}^{{2}}+{\boldsymbol{i}}\eta }d{\boldsymbol{\omega }}{\boldsymbol{^{\prime} }},$$

where P represents the principle value of integral. This method is explained in detail in ref.^30^. Optical spectra are calculated using as described in ref.^4^.

## Results and Discussion

### Structural and electronic properties

**Figure 1:**
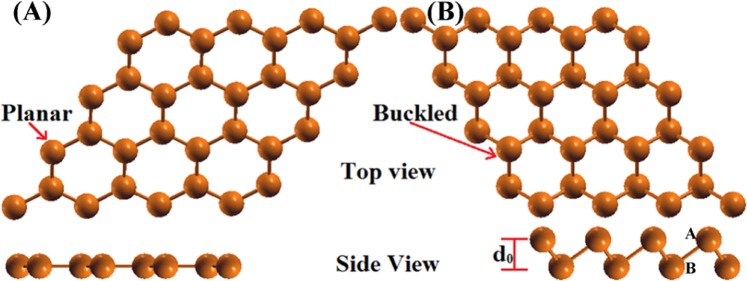
The optimized structural configurations of indiene allotropes: (**A**) planar and (**B**) buckled structures.

In this work, we have taken two atoms in the unit cell with hexagonal symmetry in both the planar and buckled allotropic modifications. The corresponding space group symmetries are P6/mmm and P3m1, respectively. The optimized structures of planar and buckled are illustrated in Fig. 1(A,B) and corresponding lattice parameters are 4.89 Å and 4.24 Å, bond lengths 2.82 Å and 3.02 Å, and angles 120° and 89.20° for the planar and buckled indiene, respectively. According to the predictions by Singh *et. al*.^23^, the planar and buckled structures of indiene allotropes are dynamically stable. The calculated cohesive energies are −2.08 eV (planar) and −2.13 eV (buckled) obtained using HSE06 functional, and it is good consistent with previous work^23^.

**Figure 2:**
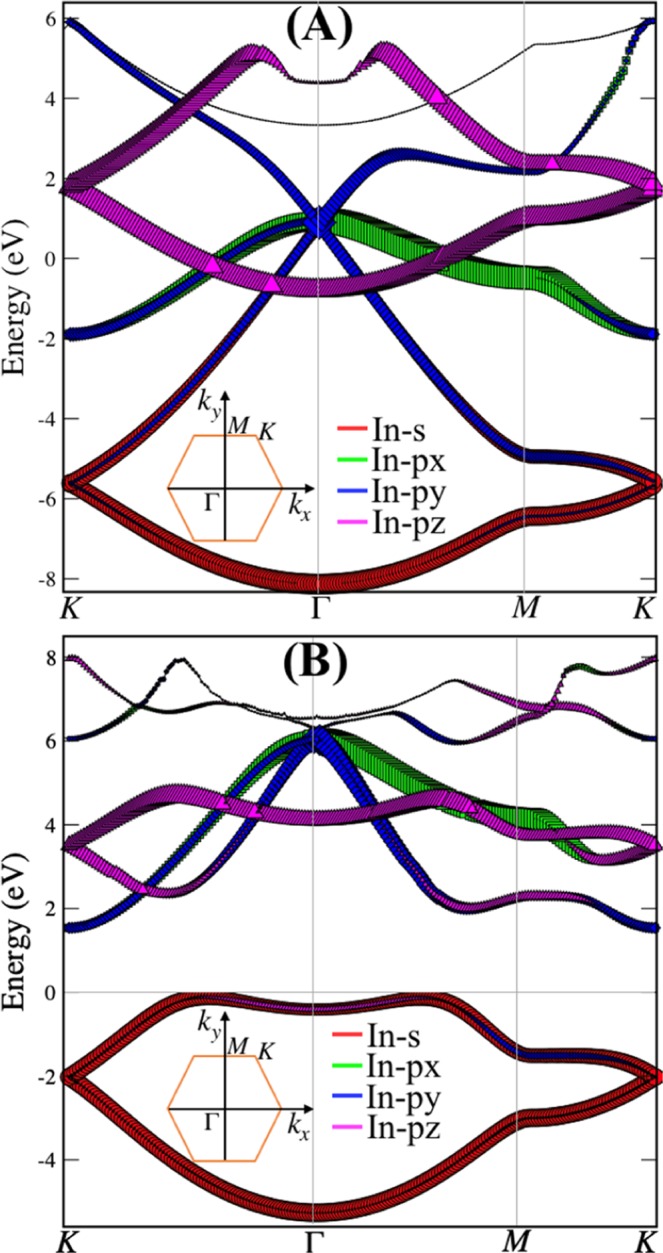
Electronic band structure, with different orbital contributions of planar (**A**) and buckled (**B**) indiene. The insets show the corresponding Brillouin zone for the hexagonal structure. p (p_x_, p_y_, p_z_) and s orbital characters of the bands are specifically distinguished using different colours and symbol sizes.

In our previous work, we discussed electronic properties of planar and buckled indiene using the PBE functional^23^. In order to more accurately predict the electronic band structure and total/projected orbital states (see Fig. S1 in ESI), and consequently the optical properties, we now use HSE06 functional which corrects the shortcoming of PBE functional regarding the band gap value (Fig. 2(A,B)). Indium has an electronic configuration of [Kr] 4d^10^ 5s^2^ 5p^1^; 4d-orbitals are completely filled and 5p-orbital has one unpaired electron. Due to the inert pair effect, there is a pertinent energy gap between 5s- and 5p-orbitals caused by fundamentally filled 4d orbital. Accordingly, making a 2D system of indium atoms utilises their outer p-orbitals without undergoing any sort of definite hybridization. These demands empty p-orbitals to form cyclic π-orbitals in addition to σ-bonds. These overlapping empty p-orbitals on individual atoms can best fit in the planar structure giving up the cyclic molecular orbitals which can be treated as holes. Thus, to gain stability, an electron from the low lying 5 s orbital takes a place in such a way that each atom is in sp^2^ hybridization configuration in planar indiene. Empty p-orbitals make empty π-clouds, which can be considered as a sea of holes, throughout the planar structures. The main contribution in the band structure comes from s- and p-orbitals.

s-orbitals make more contribution in the range between −8.15 and −4.65 eV (−5.21 and −6.10 eV) in planar (buckled) indiene, while p_x_, p_y_ and p_z_-orbitals are more contributing in the range between −0.80 and −5.13 eV (−1.50 and −7.00 eV) for planar (buckled) indiene as shown in Fig. 2(A,B), respectively. Due to sp^2^-hybridization (σ- and π-bonding), the planar structure of indiene behaves like a metal, while buckled structure shows semiconducting behaviour^23^. At the Fermi levels, σ-bonds cross two places in the electronic band structure as the dominance of p-orbitals near the Fermi levels is the main characteristic of sp^2^-hybridization, like in silicene, germanene, arsenene, and phosphorene^2,11,35,36^. Distinctive features in the band structures, like flat bands and tracking band regions, will enable the understanding of the optical properties.

### Optical properties

Imaginary part *ε*_*i*_(*ω*) of the frequency dependent complex dielectric function can be explained through the electronic band structure of a material and depicts the absorptive behaviour; the real part of dielectric function determines the dispersion effects. In our study, the exciton effects are ignored, while the local field effect is included.

**Figure 3:**
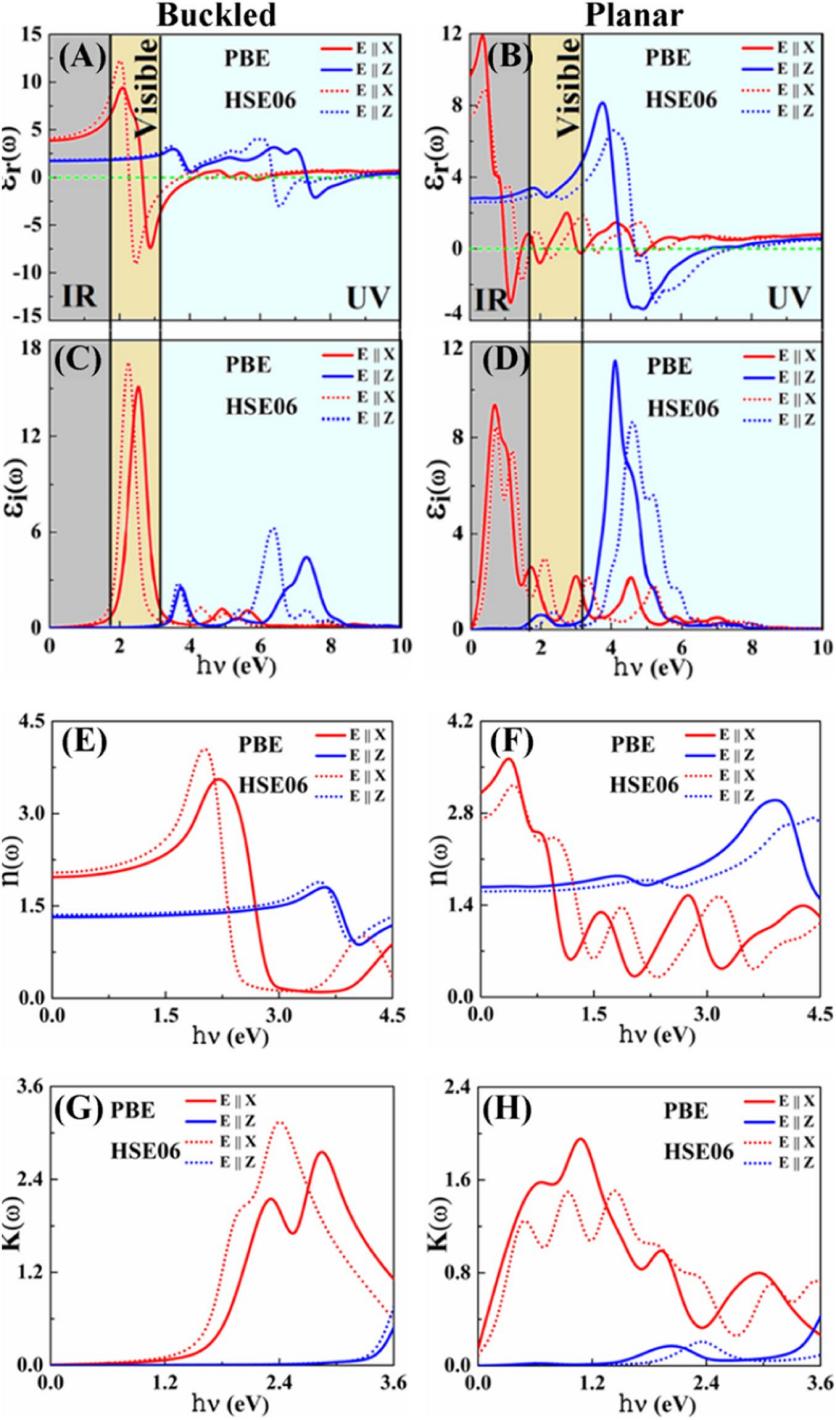
The frequency dependent optical properties simulated using the PBE and HSE06 functionals. Real part of the dielectric function of buckled (**A**) and planar **(B**) indiene and imaginary part of the dielectric function of buckled (**C**) and planar (**D**) indiene. Anisotropic behaviour of the dielectric function is obvious for both indiene allotropes. Negative values of the real parts illustrate the energy regions where indiene shows metal-like reflective behaviour regarding the incident light. The frequency dependent refractive index of buckled (**E**) and planar (**F**) indiene and extinction coefficient of buckled (**G**) and planar (**H**) indiene simulated using the PBE and HSE06 functionals. Electric field polarized parallel to indiene sheet (E||X, in-plane) is illustrated with red lines and electric field polarized perpendicular to the indiene sheet (E||Z, out-of-plane) is illustrated with blue lines.

The components of dielectric function are calculated for both in-plane (E||X) and out-of-plane (E||Z) polarizations of light (Fig. 3(A–D)). In the real part, there is an amplitude submersion – it achieves negative values in both indiene allotropes. No radiation of these energies can propagate through indiene, but is reflected from its surface, until it reaches the plasma oscillation frequency [36]: 2.36–5.60 eV (E||X) and 6.35–8.26 eV (E||Z) for the planar and 1.31–3.72 (E||X) and 4.67–7.37 eV (E||Z) for the buckled structure. Noting the static dielectric constant (dielectric constant at the zero frequency limits) one can observe an anisotropic behaviour of optical properties (Table 1), which naturally arises and is anticipated from the two-dimensional geometry of the studied structures: charge polarisation is easier in the in-plane than in the out-of-plane direction. As expected, in both allotropes the static dielectric constant is larger for the E||X polarization, an effect found in other 2D materials, like MoS_2_^37^. Since the buckled allotrope has a polarizable semiconducting band structure, its dielectric constant is higher for both polarization directions than in the planar allotrope, which is even more emphasized for the in-plane polarisation of light due to the above-mentioned reason. Regarding the constant’s value, buckled indiene could be put at the border of high dielectric constant materials^38^, with the values higher than in, for example, antimonene (ε_0_ ~ 2–5)^4^ and h-BN (ε_0_ ~ 1)^39^, similar to the one in MoS_2_ (ε_0_ ~ 1–7)^37^, while much smaller than in silicene (ε_0_ ~ 16–34)^40,41^. Higher buckling structure of indiene brings smaller overlap integrals (Equ. 1 and 2) and, consequently, smaller ε_0_ value then in silicene.

**Table 1:** Principal optical parameters obtained by DFT simulations for both in-plane (E||X) and out-of-plane (E||z) polarizations of the incoming electromagnetic wave.

Indiene allotrope	Buckled		Planar	
Polarization	E||x	E||z	E||x	E||z
Static dielectric constant	4.16	1.83	7.33	2.60
Static refractive index	2.04	1.36	2.72	1.61
Refractive index maximum (energy in eV)	4.06 (2.00)	2.27 (6.16)	3.24 (0.48)	2.71 (4.39)
Extinction coefficient maximum (energy in eV)	3.15 (2.40)	2.10 (6.50)	1.50 (0.93)	2.10 (5.21)
Plasma frequency, ω_p_ (eV)	3.66	9.27	1.71	7.45
Reflectivity maximum percentage (energy in eV)	90 (2.50)	64 (8.47)	59 (1.06)	75 (5.47)
Absorption maximum (10^6^ cm^−1^) (energy in eV)	0.35 (2.40)	0.35 (6.48)	0.17 (1.42)	0.36 (5.24)
Maximum optical conductivity energy (eV)	2.25	6.37	0.71	4.61

The energy values (in eV) at which the corresponding quantity achieves its maximal value are given in the parenthesis.

The interband transitions contributing to the imaginary part (see Fig. 3(C,D)) of the complex dielectric function are evaluated by summing over all possible transitions from occupied to unoccupied states in the electronic band structure. The peaks up to 5.60 eV are connected with π → π* transitions, while those above 5.60 eV with σ → σ* transitions, both along the M–Γ and Γ–K directions. The imaginary part of dielectric function also shows strong dependence on the polarization directions of incident light. A very interesting observation is that E||X polarization exhibits peaks in the IR (planar) and Vis (buckled) parts of the spectrum, while in the same time E||Z polarization shows strong peaks only in the UV part. This complementary behaviour of the dielectric function with regard to different parts of the electromagnetic spectrum presents a potential in applications where a polarization selective material is needed, whether it concerns reflection, absorption or transmission processes as will be commented later.

The refractive index and extinction coefficient for both in-plane and out-of-plane polarization directions of planar and buckled structure of indiene are given in Fig. 3(E–H). The values of static refractive index *n*(0) range between 1.4 and 2.7 (Table 1), while the average values at 2.25 eV (2.02 – planar and 1.05 – buckled) and 3.1 eV (0.85 – planar and 1.65 – buckled) can be compared with the bulk-case values: 1.17 and 0.7, respectively^42^. Refractive indices experience sharp drops immediately after their maxima in the respective epsilon-near-zero regions. These large changes in the refractive index (almost a 100% of the value in the case of in-plane polarization in the planar indiene) could indicate nonlinear optical regimes as found in indium tin oxide^43^. Planar indiene exhibits a distinctive maximum in the visible spectrum with a high value of 4.06, giving it an application potential as an antireflection coating in optoelectronic devices^44^, light extractor in electroluminescent devices^45^ or ultra-thin film encapsulated for photovoltaic solar cells^46^. This value is still, however, smaller than in some transition metal dichalcogenides monolayers: 6.50 (MoS_2_), 6.25 (WS_2_), 5.68 (WSe_2_) and 4.25 (MoSe_2_)^15^. On the other hand, buckling of the planar structure shifts the maximum to the IR spectrum, giving it a modulating flexibility in devices which require prevention of infra-red light or heat losses. Again, refracting property of indiene is strongly polarization dependent, as for E||Z peaks move to the UV spectrum, albeit with smaller values. Refractive index does behave more stable in the visible spectrum for E||Z polarization with almost constant values around 1.5. This value is close to the values for standard glass substrates and, based on this result, one can imagine ultra-thin dielectric mirrors with indiene component. Stacking of up to 3 layers of buckled indiene increases the value of refractive index. However, the effect is mostly bounded in the infra-red part of the spectrum^47^.

The local extinction coefficient maxima correspond to the zeroes of the real part of dielectric function (Table 1). At these energies, photons will be absorbed very fast, *i.e*. their decay path will be the shortest. Sharp peaks, thus, indicate strong absorption tendency of indiene. Since both refractive index and extinction coefficient have their maxima in the same parts of the electromagnetic spectrum, indiene allotropes are going to be opaque in those regions: planar in the visible and buckled in the infra-red and deep UV parts of the spectrum.

**Figure 4:**
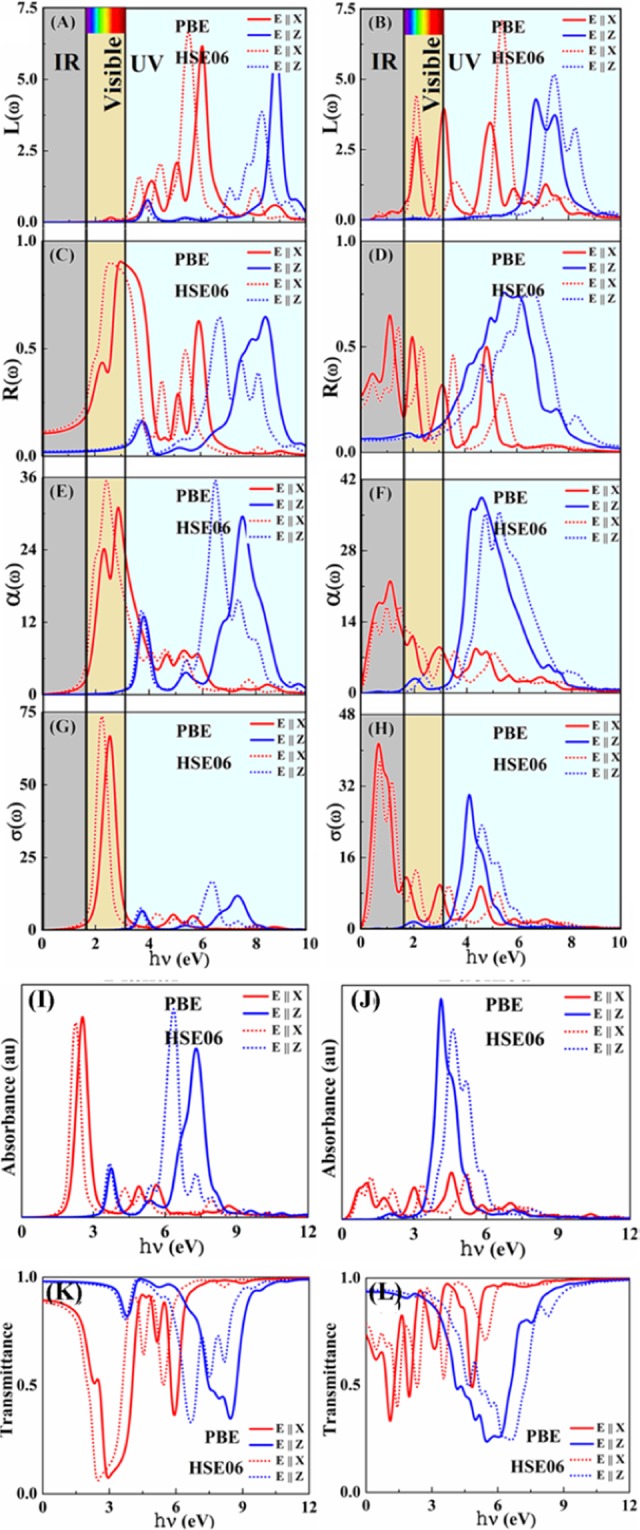
The frequency dependent energy loss spectrum, (**AB**) of buckled and planar indiene and, (**C**,**D**) reflectivity of buckled and planar indiene, absorption spectra, (**E**,**F**) of planar and buckled Indiene and, (**G**,**H**) optical conductivity of buckled and planar indiene. Absorbance (**I**,**J**) and transmittance (**K**,**L**) of buckled and planar indiene allotropes as the functions of energy, respectively. Out-of-plane polarisation shows higher transmittance in the infra-red part of the spectrum. The full line indicates the PBE functional and dotted line indicates the HSE06 functional with both directions of the electric field: electric field polarized parallel to indiene sheet (in plane, red line) and electric field polarized perpendicular to the indiene sheet (out of plane, blue line).

When a fast electron traverses a solid medium, it may loss some energy, represented by $L(\omega )$, during the excitation of other electrons in the solid. Low-energy peaks (<3 eV) appear in the buckled indiene, with respect to the planar indiene which is presented in Fig. 4(A,B), due to the separation of the valence and conduction bands and the flattening of the topmost valence band. Besides these, the most intensive in-plane plasma mode does not change significantly with the buckling of the planar structure. However, buckling does introduce another out-of-plane mode around 7.5 eV with strong intensity. In both indiene allotropes plasma frequencies for E||Z polarization is higher than for E||X polarization, as observed in other monolayers, like silicene^48^.

Rich features in the buckled indiene (buckled silicene and borophene have only two peaks^48,49^) come from the inter- and intra-bands transitions and plasma excitations, among other processes, contributing to the formation of EELS spectrum. Most of the peaks in the EELS are connected to the intra and inter-band excitations, while only the major peak corresponds to the energy of volume plasmons $\hslash {\omega }_{p}$ (Table 1). Planar and buckled indiene have two plasmon peaks for the in-plane polarization, first around 3.5 eV and 2.0 eV, which is due to the π plasmon and the other around 5.5 eV, which arises due to the π + σ plasmon for both planar and buckled indiene. Specifically, the first peak at 3.5 (2.0) eV for planar (buckled) is caused by the π → π* transition around K and M points in the band structure. On the other hand, the peak at 5.5 eV for both phases can be explained by the transition between the π → σ*/σ → π* (valence to conduction band). In the case of out-of-plane polarization direction, the two dominant peaks arise at around 4 eV (π → π* transition) and 8 eV (σ → σ* transition).

Our theoretical simulations predict very high reflectivity maxima for both indiene allotropes (Fig. 4(C,D) and Table 1). Onsets of the reflectivity peaks are aligned with the behaviour of the real part of dielectric function described earlier. Highest reflectivity of 90% is obtained in the planar indiene for the in-plane light polarization, which is achieved in the visible part of the spectrum. Very high reflectivity stands out from other mono-atomic monolayers, like borophene where it achieves the maximum of 10% in the visible part of the spectrum^49^, and grows toward the commercial optical reflectors, such as TiO_2_. This property could present the planar indiene as a potential ultra-thin reflector layer for green to violet wavelengths or as a transmission layer for red to yellow wavelengths in dichroic filters.

Strong anisotropy is also present in the reflectivity, as the light with out-of-plane polarization will experience higher reflection at much higher energies (UV part of the spectrum), an effect seen in, for example, silicene^48^. The in-plane absorption spectrum is dominated by two groups of peaks in both indiene allotropes: the first one covers the visible (IR) frequencies range starting with very intense absorptions at 2.40 eV (1.42 eV) in the planar (buckled) allotrope; the second one appears in the UV frequency range for both allotropes. The origin of the peaks at 2.40 eV (0.49 eV) and 8.73 eV (7.27 eV) is due to π → π* and σ → σ* interband transitionsbetween parallel bands in the K−Γ and Γ−M directions of the Brillouin zone (Fig. 4(E,F)) for the buckled and planar structure. The π → π* type transitions between the bands corresponding to bonding and anti-bonding states, near the Fermi energy, are liable for the peak structure in the spectrum between 2.00 and 5.60 eV (0.67 and 5.15 eV) for the buckled (planar) allotrope.

Additionally, absorption spectra show that σ → σ* electronic transitions occur at energies beyond 5.60 eV (6.38 eV) for the buckled (planar) allotrope. Actually, the lower lying valence σ states have a negligible contribution in the absorption spectrum below 5.60 eV and 6.38 eV for both allotropes. That is why only the remaining higher-occupied σ valence states contribute to the absorption beyond 5.60 eV (6.38 eV) giving rise to the 8.73 eV (7.27 eV) peak for planar and buckled structures in the spectrum. These higher-occupied states are the ones at the K and M points of the BZ and at points between them.

The absorption for the polarization parallel to the z axis (out-of-plane) is characterized by a weak intensity in the low frequency range (below 3.20 eV for both the phases). The more intense peaks appear in the frequency range beyond 3.20 eV. The origin of these peaks is due to π → σ* and/or σ → π* interband transitions according to the selection rule analysis^50^. We have found that the 3.78 eV and 4.08 eV peaks originate mainly from π → σ* electronic transitions associated with the highest occupied π band and the lowest unoccupied σ* band. On the basis of electronic band structure and the selection rules for dipole-allowed transitions^51^, we believe that 6.70 eV and 5.88 eV peaks should originate from σ → π* transitions between nearly parallel bands in the K-M regions of the BZ for both the planar and buckled allotrope. In addition, significant absorption from the visible region to the ultraviolet region can be observed in the planar indiene, while for the buckled indiene the significant absorption begins in the IR up to the visible region. So, planar and buckled indiene may become serious candidates as the saturation absorbers which can be used in laser devices or as nanoscale solar cell absorbers.

Since there are regions where the absorption has very low intensity, we calculated the transmission spectra (transmittance) as T = 1-A-R, where A and R are the absorbance and reflectance spectra (Fig. 4(I–L)). Absorbance is calculated from the absorption coefficient α as described in ref.^52^. Considering the spectral profiles of A, R and T, indiene will be an opaque material. Transparency is most present in the IR part of the spectrum. This is particularly evident for the out-of-plane polarisation of planar indiene in which it is spread into the visible spectrum. On the other hand, in-plane polarisation shows low to very low transparency in this region. This could lead to applications in devices which can benefit from polarisation selectivity, where the material has to pass the light of the specific polarisation, like Bragg filters^53^ or resonant gratings^54^.

Comparison with other two-dimensional materials points out the potential of indiene in optical applications, such as photovoltaic devices. Therefore, in the subsequent discussion we will restrict our observations to infra-red, visible and low-UV wavelengths. High absorption coefficient has been obtained around 2.4 eV in buckled indiene, as mentioned above, with a value of 3.6×10^6^ cm^−1^ (planar indiene shows 1.8 × 10^6^ cm^−1^ peak in the IR). These values are higher than in bulk indium which has the absorption coefficient of 1.05 × 10^6^ cm^−1^ at 2.25 eV and 1.07 × 10^6^ cm^−1^ at 3.1 eV^42,43^. Even more important, these values surpass the absorption coefficient of monolayer MoS_2_ (1.5 × 10^6^ cm^−1^ at 2.5 eV), which has been proposed as the sub-nanometer material with huge potential in photovoltaic systems. Furthermore, this evaluation is even more enhanced when compared to the case of graphene, which has the absorption coefficient of 0.7 × 10^6^ cm^−1^ at 2.5 eV^52^. This large value of absorption coefficient in buckled indiene comes from transitions between parallel bands which spread across almost the whole Brilloiun zone (Fig. 3). However, indiene has smaller thickness than MoS_2_, resulting in about the same absorbance percentages of 10% (Fig. 4). We propose that the puckering of the indiene structure or a multilayer homo-/hetero-structure with other similar monolayers could enhance the absorbance and bring indiene closer to photovoltaic applications. An example is the multilayers of indiene in which an increase in absorbance is demonstrated^47^. The effect is especially pronounced in the buckled allotrope, along with the blue shift of absorption peaks toward the visible spectrum. Other modifications of two-dimensional indiene allotropes are a subject of an on-going research.

The calculated real part of optical conductivity spectra $\sigma (\omega )$ obtained from the electronic band structure corresponding to the imaginary part of the dielectric function is shown in Fig. 4G,H for the buckled and planar indiene, respectively. It is calculated as $\sigma (\omega )=\frac{\omega {\varepsilon }_{i}(\omega )}{4\pi }$, where $\omega$ is the frequency in cm^−1^. Optical conductivity is zero below 1.00 eV (Eǁx) and 3.50 eV (Eǁz) for the buckled, and below 0.24 eV (Eǁx) and 1.65 eV (Eǁz) for the planar allotrope. The maximum optical conductivity for in-plane corresponds to energy of 2.25/0.71 eV and for out-of-plane corresponds to energy of 6.37/4.61 eV for buckled (planar) indiene, respectively, which coincide with the absorption maxima. The maximal conductivity greatly surpasses the ones of MoS_2_, where it peaks at about 2.90 eV^55^, and silicene, where it peaks in the UV spectrum^56^. These values support the conceivable utilization of indiene allotropes in opto-electronic gadgets as outlined earlier.

### Effects of external electric field

In the present study, the transverse electric field is applied on the indiene sheet. Due to the linear dispersion behaviours of π and π* band lines between the K-Γ and Γ-M points, the effective charge carriers in planar indiene are massless fermions. In this case, the Hamiltonian for electronic band of planar structure of indiene around the Dirac points, like that of graphene^57^, is defined by:

$$(\begin{array}{cc}\Delta  & \hslash {v}_{f}({k}_{x}-i{k}_{y})\\ \hslash {v}_{f}({k}_{x}+i{k}_{y}) & -\Delta \end{array}),$$

where *k* represents the momentum and *v*_*f*_ is the Fermi velocity of effective charge carriers near the Dirac points. Also, $\varDelta$ represents the onsite energy difference between the indium atoms in the unit cell containing two atoms. Here, we can take the value zero ($\Delta =0$) due to the existence of inversion symmetry and which follows the linear dispersion near the Dirac point, i.e. $E=\pm \hslash {v}_{f}|k|$. In some cases, value of Δ becomes finite as well as dispersive nature and it follows near the Dirac point $E=\pm \sqrt{{\Delta }^{2}+{(\hslash {v}_{f}k)}^{2}}$, when the breaking of inversion symmetry such as buckling case. Actually, the electric field creates a potential difference on atoms which are not in a similar sub-plane on account of the buckled morphology and, along these lines, moves in the energy bands. In this manner, a recognizable impact can be normal just in the buckled allotrope of indiene.

**Figure 5:**
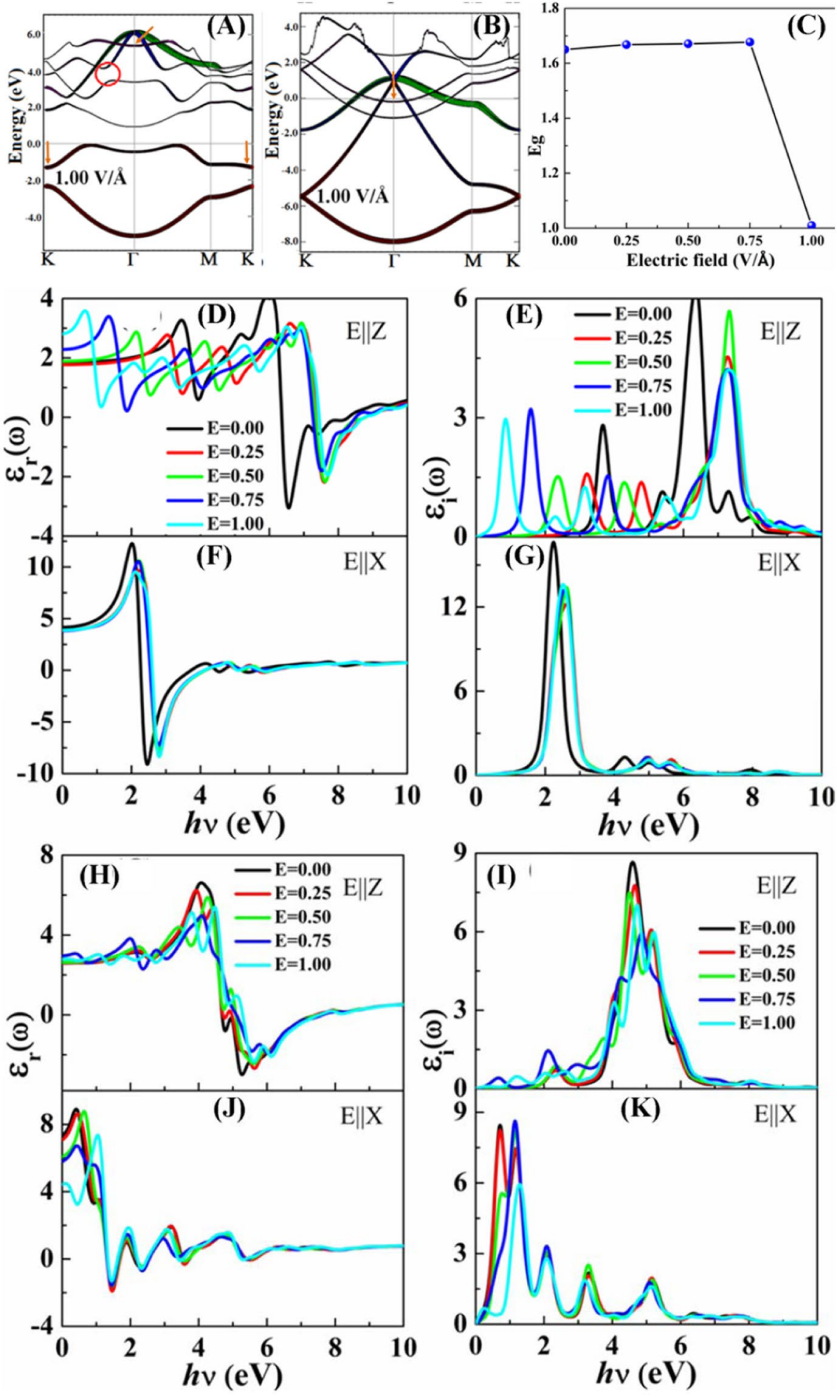
Electronic band structure (**A**) buckled and (**B**) planar indiene at 1 V/Ǻ applied external electric field. Orange arrows denote the shifts of respective conduction and valence bands. Circles point to the pseudo-gap opening in the conduction band. Variation of the energy band gap (**C**) with the applied external electric field in buckled indiene. Frequency dependent complex dielectric function for different polarization direction out-of-plane (EǁZ) and in-plane (EǁX) (**D**,**F**) real part, (**E**,**G**) imaginary part of dielectric functions of buckled indiene and (**H**,**J**) real part, (**I**,**K**) imaginary part of dielectric function of planar indiene with applied external electric field, respectively.

Applying a perpendicular electric field is a viable approach to specifically control the energy band gap, as was shown for other mono-elemental monolayers, such as arsenene^35^, silicene^36^ and stanene^58^. In the case of buckled indiene allotrope, a division exists between A and B sublattices. It is a fascinating issue to inspect how the electronic band structure is adjusted under an applied electric field. As discussed in the previous work, under mechanical strain, buckled structure of indiene remains an indirect semiconductor^23^. Under applied external electric field, it keeps its semiconducting behaviour up to 1 V/Å (Fig. 5(A)). We can observe, as in the planar case, a conduction band shift toward the Fermi level. However, a shift, albeit upward, occurs in the valence band as well, specifically at the K point. These two effects contribute to the changes of the band gap with increasing electric field (Fig. 5(A)). As the shifts are most pronounced at higher electric field strengths, a sharp drop in the band gap size occurs close to 1 V/Å as shown in Fig. 5. Another observed effect in the conduction band is an increasing opening between the conduction bands (represented with orange circle in Fig. 5(A) and see Fig. S2 in ESI). We conjecture that the most significant changes of the p_z_ orbital energies do not happen until the out-of-plane force exerted by the external field overcomes the electrostatic forces from other in-plane orbitals. From Fig. 5(A) at such higher electric field strengths, the atoms are more polarised in the out-of-plane direction as presented in Table 2 and corresponding electronic band lines are more degenerate at K-point. In the conduction band minimum, p_z_-orbital is the main contribution reducing the band gap. But indirect band gap is shifted from Γ-K to K-Γ.

**Table 2:** Static dielectric functions at different external electric fields for both indiene allotropes.

	Electric field (V/Å)					
		0.00	0.25	0.50	0.75	1.00
In buckled	E||x	4.16	3.86	3.85	3.83	3.88
	E||z	1.83	1.77	1.89	2.28	2.81
In planar	E||x	7.33	7.07	6.08	5.79	4.42
	E||z	2.60	2.59	2.63	2.95	2.79

We have analysed dependence of the electric field strength on indiene monolayer ranging from 0 to 1 V/Å see Fig. S2 in ESI. Expectedly, the electronic band structure of planar indiene shows no changes. Its metallic behaviour is preserved even after applying 1 V/Å electric field. However, one conduction band, represented by an arrow in Fig. 5(B), does shift symmetrically towards the Fermi level. This band, as expected, corresponds to the out-of-plane oriented p_z_ orbital. From Fig. 5(D–K), it is evident that transverse static electric field does significantly affect the real and imaginary parts of dielectric function of planar indiene for out-of-plane polarisation of light. The peak positions are shifted by approximately 1 eV, while their intensities are somewhat decreased. This will have three effects: (i) the region where metallic-like reflection occurs, defined by ε_r_ < 0, shifts to higher energies, (ii) high energy absorption peak, originating from the ε_i_ maximum, is blue-shifted, and (iii) low energy absorption peak is red-shifted by almost 3 eV, from UV (at zero field), through visible (at 0.75 V/Å field), to infra-red spectrum (at 1.00 V/Å field). The low energy absorption peak shift can be explained by observing the changes induced by the field on the electronic band structure (Figs 5 and S2). Under large electric fields, p_z_ orbitals shift down in energy by about 3 eV, contributing significantly to the absorption process at lower energies via larger joint density of states. Blue-shift of the high energy peak cannot be so easily explained. We suppose that the reason is the removal of degeneracy of p-states at the Γ point caused by the electric field (Fig. S2). This lessens their contribution to the DOS and absorption band intensity. Meanwhile, the higher energy conduction bands at K point are almost insensitive to the influence of electric field. They could have a dominant contribution to the absorption band at around 7.4 eV. A significant shift downward of those levels occurs at 1 V/ Å, but it is compensated by the splitting of s-states at K point around −2 eV. In the end, these effects demonstrate that external electric field can be effectively used to modify the absorption of buckled indium monolayer. Static dielectric constant, ε_r_(0), also experiences an increase, from 1.83 (at zero field) to 2.81 (at 1.00 V/Å field) (Table 2). The change is much less for the in-plane polarisation of light. With the increasing the strength of fields, the interband transitions start from lower photon energy to higher photon energy up to 10 eV as compare to without effect of electric field and also enhanced the absorption strength of indiene monolayer at higher photon energy. While, the strong influence of the electric field is seen for the static dielectric constant of planar indiene for in-plane polarisation. It decreases from 7.33 to 4.42 with increasing field strength. In the case of planar indiene, the transverse static electric field does not significantly affect the dielectric function peaks in both polarization directions.

## Conclusions

Since indium monolayers have still not yet been synthesized, *ab initio* theoretical simulations are currently the only tool available to assess their properties. Our simulations indicate that their properties can be beneficial in different application areas with emphasis on the optical applications. Their electronic properties can be strain engineered, while in this work it is shown that they manifest diverse optical behaviour. Large optical anisotropy is observed for the in-plane and out-of-plane light polarizations. This can be very useful in ultra-thin optical polarisers based on indium, for infra-red, visible and ultra-violet light. Using monolayer materials as light filters can have many advantages over their bulk counterparts, due to their morphological and size related advantages. As seen from this and previous works, monolayers and their respective properties are readily modified by strains, electric fields, surface functionalization and doping. Additionally, they possess flexibility which can be exploited on free-shape surfaces, like vehicles, buildings, clothes and skin. Among the optical properties, very high reflectivity in the visible spectrum of planar indiene is distinguished. Further development can be two-fold directed. For ultra-thin reflector devices, one could extend this high reflectivity to lower visible wavelengths. This is a part of the on-going work. Also, reflectivity in the buckled indiene is characterised by the almost equally spaced reflectance peaks, which span the segment from the infra-red to the ultra-violet wavelengths. Equalizing their separation and reflectance percentage could introduce indiene to applications in wavelength selective reflectors. On the other hand, for ultra-thin absorption devices, like solar radiation absorbers, one could strive to supress the reflectivity and allow the high absorptive properties to gain dominance. Minimal reflectance requires the refractive index as close as possible to 1, a condition fulfilled in both allotropes for the out-of-plane polarization. However, for the in-plane polarization, one would have to shift the plasma frequency toward the low energy visible or NIR spectrum. Upon achieving the refractive index close to 1, further reduction of reflectance would not be effective, as it would require reducing the extinction coefficient, which would simultaneously reduce the absorbance.

In this work, we have also studied the effect of transverse static electric field on the electronic and optical properties of planar and buckled indiene. We have shown that the planar structure remains metallic, while the band gap in the buckled structure can be significantly changed by applying the external electric field. We observed plausible changes in the dielectric function due to the band line and band gap changes. These insights provide a theoretical basis for the design and applications of planar and buckled indiene in opto-electronic materials.

Indiene-based optical device’s functionality can be effectively modified by imposing the buckling of the monolayer structure, which should by itself be pervious to the substrate choice. This will be proved in our future work. On the other hand, the simulated reflectance, absorbance and EELS spectra clearly show that planar and buckled indiene allotropes can be differentiated using these spectroscopies due to the calculated peak positions. We have, thus, also provided the grounds for possible characterisation of indiene allotropic modifications using spectroscopy methods.

## Supplementary information

Electronic Supplementary Information (ESI)
