# Supplementary material for: Effect of electric field on optoelectronic properties of indiene monolayer for photoelectric nanodevices
Source: Sci Rep. 2019 Nov 21;9:17300. doi: 10.1038/s41598-019-53631-2 (PMC6872639; doi:10.1038/s41598-019-53631-2)
Supplement: Supplementary file 1 — Electronic Supplementary Information (ESI) [file 41598_2019_53631_MOESM1_ESM.docx]

**Electronic Supplementary Information (ESI)**

**Effect of electric field on optoelectronic properties of indiene monolayer for photoelectric nanodevices**

Deobrat Singh^1^, Sanjeev K. Gupta^2,*^ Igor Lukačević^3^, Matko Mužević^3^, Yogesh Sonvane^4^_,_  and Rajeev Ahuja^1,5^

^1^Condensed Matter Theory Group, Department of Physics and Astronomy, Uppsala University, Box 516, 77120, Uppsala, Sweden

^2^Computational Materials and Nanoscience Group, Department of Physics, St. Xavier's College, Ahmedabad 380009, India

^3^Department of Physics, Josip Juraj Strossmayer University of Osijek, 31000 Osijek, Croatia

^4^Advanced Material Lab, Department of Applied Physics, S.V. National Institute of Technology, Surat, India

^5^Applied Materials, Department of Materials and Engineering, Royal Institute of Technology (KTH), S-100 44 Stockholm, Sweden

Corresponding authors: [sanjeev.gupta@sxca.edu.in](mailto:sanjeev.gupta@sxca.edu.in) (Dr. Sanjeev K. Gupta)


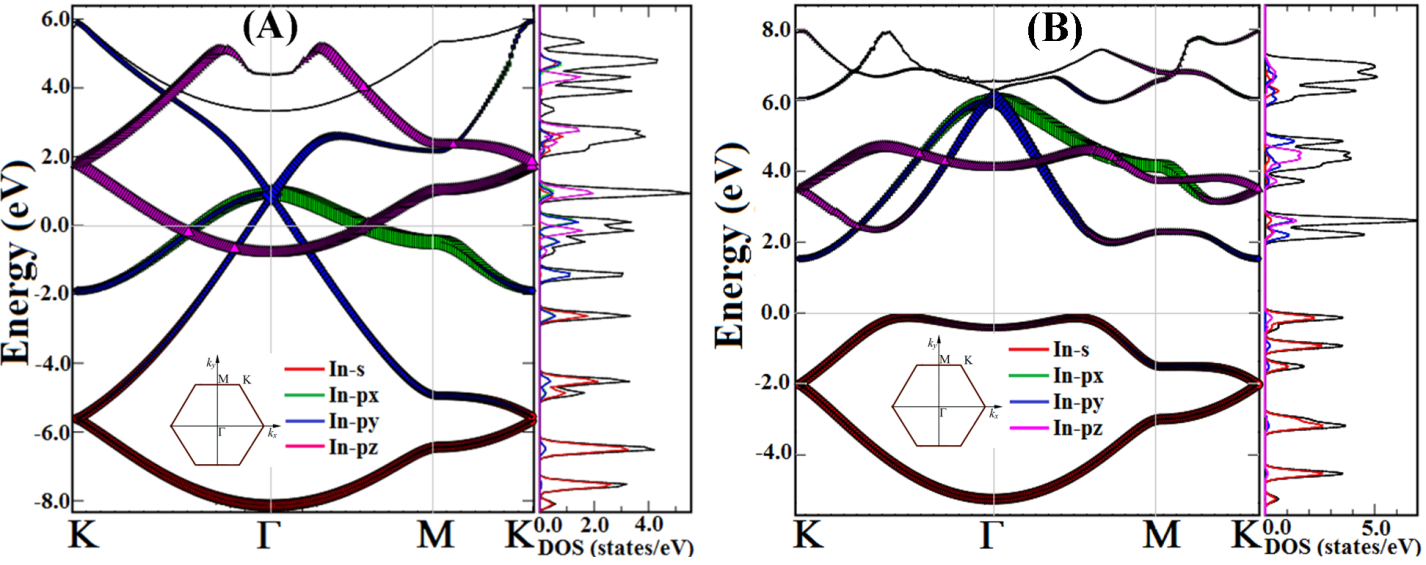


**Figure S1.** Electronic band structure, partial density of states with different orbital contributions in the band structure of planar (A) and buckled (B) indiene. The insets show the corresponding Brillouin zone for the hexagonal structure. The main contributions of the orbitals have p (p_x_, p_y_, p_z_) and s characters of the bands are specifically distinguished using different colours and symbol sizes. Larger symbols indicate larger contributions to the density of states.

**
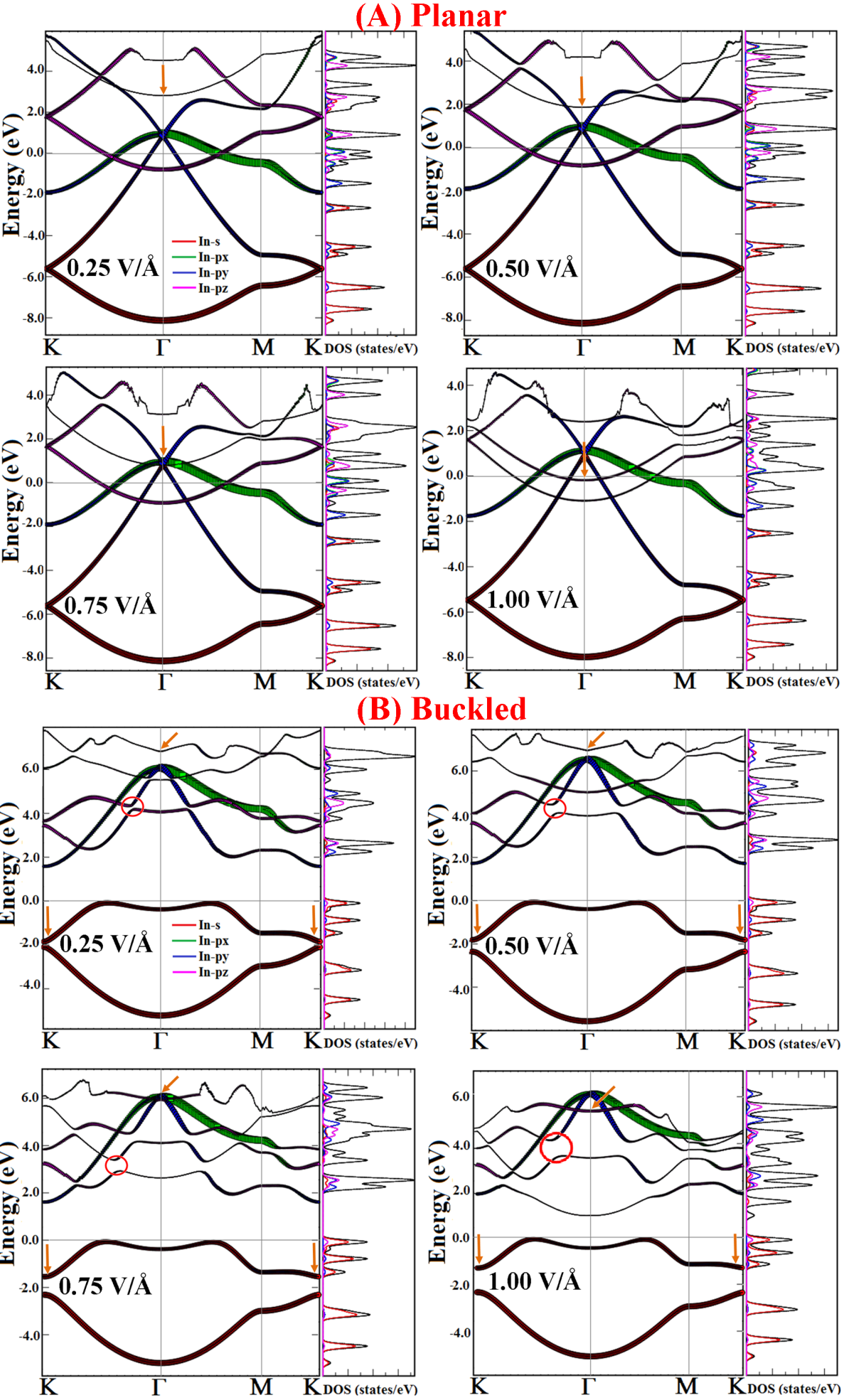
**

**Figure S2.** Electronic band structure, total and projected DOS of (A) planar indiene (B) Buckled indiene for different values of applied external electric field. Orange arrows denote the shifts of respective conduction and valence bands. Circles point to the pseudo-gap opening in the conduction band.
